# Comparative Analysis of Quinolone Resistance in Clinical Isolates of *Klebsiella pneumoniae* and *Escherichia coli* from Chinese Children and Adults

**DOI:** 10.1155/2015/168292

**Published:** 2015-02-10

**Authors:** Ying Huang, James O. Ogutu, Jiarui Gu, Fengshu Ding, Yuhong You, Yan Huo, Hong Zhao, Wenjing Li, Zhiwei Zhang, Wenli Zhang, Xiaobei Chen, Yingmei Fu, Fengmin Zhang

**Affiliations:** ^1^Department of Microbiology, Pathogenic Biology, Harbin Medical University, Harbin, Heilongjiang 150081, China; ^2^Heilongjiang Provincial Key Laboratory of Immunity and Infection, Harbin, Heilongjiang 150081, China; ^3^Harbin Children's Hospital, Harbin, Heilongjiang 150081, China; ^4^Department of Laboratory Diagnosis, Heilongjiang Provincial Hospital, Harbin, Heilongjiang 150081, China

## Abstract

The objective of this study was to compare quinolone resistance and *gyrA* mutations in clinical isolates of *Klebsiella pneumoniae* and *Escherichia coli* from Chinese adults who used quinolone in the preceding month and children without any known history of quinolone administration. The antimicrobial susceptibilities of 61 isolates from children and 79 isolates from adults were determined. The mutations in the quinolone resistance-determining regions in *gyrA* gene were detected by PCR and DNA sequencing. Fluoroquinolone resistance and types of *gyrA* mutations in isolates from children and adults were compared and statistically analyzed. No significant differences were detected in the resistance rates of ciprofloxacin and levofloxacin between children and adults among isolates of the two species (all *P* > 0.05). The double mutation Ser83→Leu + Asp87→Asn in the ciprofloxacin-resistant isolates occurred in 73.7% isolates from the children and 67.9% from the adults, respectively (*P* = 0.5444). Children with no known history of quinolone administration were found to carry fluoroquinolone-resistant *Enterobacteriaceae* isolates. The occurrence of ciprofloxacin resistance and the major types of *gyrA* mutations in the isolates from the children were similar to those from adults. The results indicate that precautions should be taken on environmental issues resulting from widespread transmission of quinolone resistance.

## 1. Introduction

It is widely accepted that overuse of antibiotics in hospitals and communities is one of the major contributing factors to bacterial resistance [1]. However, it is worth noting that decreasing antibiotic use does not necessarily directly correspond to complete eradication of antimicrobial resistance in bacterial populations [2, 3]. It has been reported that drug resistance and its determinants could persist in the bacterium even in the absence of the corresponding antibiotics [1]. Furthermore, it has recently been shown that quinolone resistance also develops in the absence of antibiotic selective pressure [4, 5]. Therefore, we may postulate that the existence of antimicrobial-resistant isolates from clinical cases may not necessarily result from antimicrobial use.

Fluoroquinolones have been widely used in the treatment of a variety of infections due to their broader antimicrobial spectra and improved pharmacokinetic properties. However, the problem of quinolone resistance is becoming increasingly serious with the extensive use of the agents [6, 7].* Klebsiella pneumoniae* and* Escherichia coli *are the two most common quinolone-resistant* Enterobacteriaceae*. Resistance to quinolones in enterobacteria is mainly due to mutations in the quinolone target enzymes (DNA gyrase and topoisomerase IV) encoded by* gyrA* and* parC *[8, 9]. Besides, plasmid-mediated quinolone resistance genes also confer low levels of quinolone resistance [10].

Due to their adverse effects on children's cartilage, the prescription of quinolones has been restricted from pediatric cases [11]. Thus, children can be considered as a population lacking exposure to quinolones. With the purpose of investigating drug resistance among people with and without the history of fluoroquinolone administration within the same area, we collected the clinical isolates of* K. pneumoniae *and* E. coli* from both preschool children, who have no known history of quinolone administration, and adults, who used quinolone in the preceding month of the sample collection, in Harbin, Heilongjiang, China. Fluoroquinolone resistance and types of* gyrA *mutation in the clinical isolates were detected and compared between the two groups.

## 2. Materials and Methods

### 2.1. Bacterial Collection

During the period of March to December 2009, 77* K. pneumoniae* and 63* E. coli* nonrepetitive isolates were consecutively collected from three tertiary hospitals in Harbin city, the provincial headquarters of Heilongjiang province, Northeast China. Seventy-nine isolates came from adult patients while sixty-one isolates from children, comprising 21 infants (<1 year), 20 toddlers (1–3 years), and 20 preschoolers (3–6 years). The strains were isolated from stool, nasopharyngeal swabs, and sputum samples of in-patients in surgical wards, internal medicine wards, intensive care units, and pediatric wards. A questionnaire surveyed information about quinolone usage in children and adults. The clinical strains were obtained only from children without the history of quinolone administration and adults who had taken quinolones in the four weeks preceding specimen collection. All the specimen providers or the children's guardians agreed to participate in the research projects and signed the informed consent. The Institute Research Board of Harbin Medical University checked and approved the study (Report number HMUIRB20140009).

Identification of the bacteria to species level was done through the API 20E system (BioMérieux, Marcy-I'Etoile, France). All isolates were confirmed as nonreplicate strains by Randomly Amplified Polymorphism DNA (RAPD) analysis (data not shown).

### 2.2. Antimicrobial Susceptibility Test

Minimum Inhibitory Concentrations (MICs) of ciprofloxacin were determined by the microdilution method according to Clinical and Laboratory Standards Institute (CLSI) guidelines [12]. The susceptibility to other antimicrobial agents was determined using Kirby-Bauer disk diffusion test with antimicrobial agent discs (OXOID, UK) following the CLSI guidelines [13].* E. coli* ATCC 25922 and* K. pneumoniae *ATCC 10031 were used as means of quality control.

Isolates with reduced susceptibility to third generation cephalosporins were subjected to phenotypic detection of ESBL production by using cefotaxime (CTX) and ceftazidime (CAZ) discs and double-disk synergy (DDS) method as recommended by the Clinical and Laboratory Standards Institute [14]. AmpC production was detected using cefoxitin alone and in combination with boronic acid and confirmation was done by three-dimensional disk method. Isolates with reduced susceptibility to cefoxitin were subjected to a modified three-dimensional extract test to confirm AmpC production [15].

### 2.3. Amplification and Sequencing of the* gyrA* Gene Fragments

All isolates of* K. pneumoniae* and* E. coli *were used to determine the DNA sequence of QRDR of* gyrA*. Oligonucleotide primers used for amplification of fragments encompassing the QRDR were* gyrA*-F (5′-TGCGAGAGAAATTACACC-3′) and* gyrA*-R (5′-AATATGTTCCATCAGCCC-3′). The* gyrA *gene fragments were amplified from crude cell lysates. PCR products were purified and then subjected to bidirectional sequencing using an automated DNA sequencer (ABI PRISM 373; Applied Biosystems, Foster City, CA) with the same primers used in the PCR amplification. The nucleotide sequences and the deduced amino acids were compared to that of* K. pneumoniae* ATCC 13883 (GenBank DQ673325) and* E. coli *K-12 (GenBank NC_010473.1) using the online ClustalW2 multiple sequence alignment program. All the experiments were performed in accordance with relevant guidelines and regulations. The partial sequences of the variant* gyrA *genes in the clinical isolates were submitted to GenBank under accession numbers EU430280 through EU430289 and JQ694717 through JQ694722.

### 2.4. Statistical Methods

Resistance rates among the isolates were analyzed with paired chi-square test. Comparisons of fluoroquinolone resistance and individual GyrA alterations between adults and children of all age groups were analyzed by Fisher's exact test or Pearson chi-square test (IBM SPSS 16.0 statistical package). *P* < 0.05 was considered as statistically significant.

## 3. Results

### 3.1. Antimicrobial Resistance in* K. pneumoniae *and* E. coli* Isolates

A comparison of antimicrobial agent susceptibility between isolates from children and adults in* K. pneumoniae* and* E. coli *was shown in Table 1. Among isolates of the two species, there were no significant differences in the resistance rates of ciprofloxacin and levofloxacin between children and adults (all *P* > 0.05). In* K. pneumoniae*, the MIC_50_, MIC_90_, and MIC range for ciprofloxacin in isolates from children and adults were 8 mg/L versus 2 mg/L, 256 mg/L versus 256 mg/L, and 0.5–256 mg/L versus 0.5–512 mg/L, respectively. In* E. coli* isolates from children and adults, the ciprofloxacin MIC_50_ was 64 mg/L versus 64 mg/L, MIC_90_ was 128 mg/L versus 256 mg/L, and MIC range was 0.5–512 mg/L versus 1–512 mg/L. The prevalence of ciprofloxacin resistance in children varied with the age. Resistance to ciprofloxacin was found in 37.5% of the isolates from infants aged under 6 months, which was lower than the percentage observed in the other age groups (69.23% of 6-month to 1-year infants, 70.0% of 1- to 3-year toddlers, and 60.0% of 3- to 6-year preschool children).

As shown in Table 1, resistance rates to other classes of antimicrobial agents in* E. coli* isolated from children and adults were quite similar (all *P* > 0.05). However, in* K. pneumoniae*, the resistance rates for cefotaxime, ceftriaxone, and ceftazidime in children were statistically higher than those in adults (all *P* < 0.05). Occurrences of the production of extended-spectrum beta-lactamases (ESBLs) isolates from children and adults were 57.9% and 38.5%, respectively. In contrast, cefoxitin resistance rate was significantly lower in the isolates from children (*P* = 0.0085). Of note, the occurrence of AmpC in children was 7.9%, which is similar to that in adults (15.4%, the Fisher exact test *P* = 0.4806). The amikacin resistance rate of* K. pneumoniae* in children is also higher than that of adults (*P* < 0.05). Among the resistant isolates from adults, 9 (23.1%) were found an absence of inhibition zone around the amikacin disc (diameter <6 mm), while 25 (65.8%) of the isolates from children were observed to have this phenomenon (*χ* ^2^ = 14.2398, *P* = 0.0002, Table 1).

### 3.2. Resistance to Ciprofloxacin and* gyrA* Mutations in Isolates from Children and Adults

All the 140 isolates were investigated for QRDR mutations of* gyrA*. 46 of the 61 isolates (75.4%) from children were found to contain* gyrA* mutations. A similar prevalence pattern of the* gyrA* mutation was also observed in the isolates of adult origin (64/79, 81.0%, *P* = 0.43). Among the 38 isolates resistant to ciprofloxacin from children, 37 (97.4%, 37/38) were found to contain the* gyrA* mutation, which is quite similar to the prevalence in isolates from adults (55/56, 98.2%, *P* = 1.00).

As shown in Table 2, the double mutation Ser83→Leu + Asp87→Asn was the most prevalent type of GyrA changes in antimicrobial-resistant isolates from both children and adults (73.7% versus 67.9%, *P* = 0.5444). The single mutation Ser83→Ile was not detected in the isolates from children but was detected in 19.6% of the isolates from adults (*P* = 0.0026). The single mutation Ser83→Leu was detected in 18.4% of the isolates from children but was not found in those from adults (*P* = 0.0012, Table 2). GryA mutation was also found in 18 isolates nonresistant to ciprofloxacin, including 9 isolates from children and 9 isolates from adults. The mutation patterns were listed in Table 2.

### 3.3. GyrA Mutations in Children with Different Age Groups

The 61 isolates from children were assigned into 4 groups according to physiological development and participation in social activities, namely, young infants (<6 months), infants (6–12 months), toddlers (1–3 years), and preschoolers (age of 3–6 years). It was found that the mutations Ser83→Leu + Asp87→Asn, Ser83→Leu, Ser83→Thr, and Asp87→Asn were the common types of mutations in GyrA, and Ser83→Leu + Asp87→Asn was the most prevalent double mutation. In addition, there was no significant difference in terms of individual types of mutation in GyrA in children among different age groups (*P* > 0.05).

## 4. Discussion

There is a growing concern over antibiotics resistance as a result of antibiotics overuse in hospital and communities. Thus, restriction on the use of antibiotics has become one of the most important measures for control emergence and spread of drug-resistant bacteria. This study involves clinical isolates obtained from children in the absence of fluoroquinolone usage history, as quinolones are not recommended for pediatric use due to the adverse effects on joints. However, our findings showed that children harbored quinolone-resistant isolates being similarly resistant to ciprofloxacin and levofloxacin compared with the isolates from adults. Moreover, isolates from children were found to have similarly high ciprofloxacin MIC levels with those from adults. By sequencing the QRDRs of* gyrA*, we found that the double mutation Ser83→Leu + Asp87→Asn was similarly predominant among the quinolone-resistant isolates from both children and adults.

A previous study conducted in Spain found 5% resistance rate of ciprofloxacin in* E. coli* isolates from healthy children [16]. Other studies have also recorded quinolone-resistant* Enterobacteriaceae* from children in recent years [17, 18].* GyrA* mutations involved in quinolone resistance in isolates from infants were most recently discovered in Spain [19]. The resistance rate to ciprofloxacin in children was up to 22% in these regions [17]. Compared to these regions, the ciprofloxacin resistance rate and the resistant level (MIC) found in the present study are notably higher. In spite of the difference in resistance rate and resistant level, the findings in the present study and others demonstrated the quinolone resistance and the involved mutation mechanisms in nonexposed population under high antibiotic pressure. It indicates that drug resistance may be transmitted and persist in people who are not exposed to the corresponding antibiotics.

Various reports on the different aspects of resistance have revealed that persistence of resistance in human or animal populations relies more on the transmission of resistance in the public environment than on drug pressure [5, 20]. Drug-resistant isolates of bacteria were detected even in remote mountainous areas of Amazon where antibiotics have never been used [4]. Presence of drug-resistant isolates in children might be attributed to their increased contact with adults and playmates in families, daycare, or school settings [21, 22]. Among the environmental factors, food supply chain may be the major source of antibiotic resistance in “naïve” humans as an indirect consequence of overuse of antibiotics for curative and preventative purpose in stock farming and agricultural products. In 2000, the use of antibiotics in raising animals was questioned [23]. Shortly after, antibiotic-resistant* E. coli* was detected in poultry products [24]. The situation of antibiotic-resistant bacteria from food chain sources has become a notable societal issue which should be worthy of attention [25–29]. Furthermore, pollution by antibiotic resistance genes from stock farming and antibiotics in manure and metabolites could increase the chances of resistance acquisition by human pathogens [30].

In summary, our data showed similar prevalence of quinolone resistance and type of* gyrA* gene mutation in* E. coli* and* K. pneumoniae* among children and adults. The conclusion that there exists similarity in fluoroquinolone resistance rates between children and adults in a region under high community antibiotic pressure constrains us to pay close attention to the environmental and community reservoirs of resistance and take precautions to prevent perpetuating antibiotic-resistant isolates and their transmission and dissemination.

## Figures and Tables

**Table 1:** Resistance of 140 *K. pneumoniae* and *E. coli* isolates to antimicrobial agents in children and adults.

Antimicrobial agent	*K*. *pneumonia* (*n* = 77)				*E*. *coli* (*n* = 63)			
	Children	Adults	*P* value	OR 95% CL	Children	Adults	*P* value	OR 95% CL
	(*n* = 38)	(*n* = 39)			(*n* = 23)	(*n* = 40)		
Ciprofloxacin	60.5 (23)	69.2 (27)	0.4235	(0.2659, 1.7464)	65.2 (15)	72.5 (29)	0.5443	(0.2359, 2.1443)
Levofloxacin	34.2 (13)	33.3 (13)	0.9351	(0.4044, 2.6748)	43.5 (10)	65.0 (26)	0.0965	(0.1450, 1.1832)
Cefoxitin	10.5 (4)	35.9 (14)	0.0085^*^	(0.0617, 0.7154)	4.3 (1)	12.5 (5)	0.4022	(0.0348, 2.9070)
Cefotaxime	76.3 (29)	46.2 (18)	0.0067^*^	(1.4143, 9.9920)	52.2 (12)	52.5 (21)	0.9801	(0.3535, 2.7560)
Ceftriaxone	71.1 (27)	48.7 (19)	0.0457^*^	(1.0081, 6.6219)	56.5 (13)	52.5 (21)	0.7578	(0.4191, 3.3007
Cefoperazone	50.0 (19)	51.3 (20)	0.9104	(0.3887, 2.3218)	65.2 (15)	55.0 (22)	0.4277	(0.5131, 4.4294)
Ceftazidime	71.1 (27)	41.0 (16)	0.008^*^	(1.3674, 9.1048)	13.0 (3)	25.0 (10)	0.3417	(0.1100, 1.8410)
Cefepime	21.1 (8)	15.4 (6)	0.5191	(0.4560, 4.7175)	26.1 (6)	25.0 (10)	0.9042	(0.3273, 3.4254)
Aztreonam	63.2 (24)	43.6 (17)	0.0853	(0.8896, 5.5324)	30.4 (7)	37.5 (15)	0.5712	(0.2440, 2.1792)
Gentamicin	57.9 (22)	43.6 (17)	0.2094	(0.7213, 4.3895)	60.9 (14)	77.5 (31)	0.1595	(0.1475, 1.3826)
Amikacin	76.3 (29)	38.5 (15)	0.0008^*^	(1.9202, 13.8422)	4.3 (1)	17.5 (7)	0.2394	(0.0246, 1.8648)
Amoxycillin/Clavulanate	18.4 (7)	30.8 (12)	0.2089	(0.1751, 1.4744)	13.0 (3)	5.0 (2)	0.3453	(0.4395, 18.4792)
Piperacillin/Tazobactam	5.3 (2)	5.1 (2)	1.0000	(0.1373, 7.6933)	8.7 (2)	10.0 (4)	1.0000	(0.1445, 5.0861)

^*^ *P* < 0.05.

**Table 2:** GyrA mutations in *Enterobacteriaceae* isolated from children and adults.

Mutation	CIP-resistant strains			CIP-nonresistant strains		
	(*n* = 94)			(*n* = 46)		
	Children%	Adults%	*P* value	Children%	Adults%	*P *value
	(*n* = 38)	(*n* = 56)		(*n* = 23)	(*n* = 23)	
Ser83 → Leu + Asp87 → Asn	73.7 (28)	67.9 (38)	0.5444	21.7 (5)	8.7 (2)	0.4140
Ser83 → Leu	18.4 (7)	0 (0)	0.0012^*^	17.4 (4)	13.0 (3)	1.0000
Ser83 → Ile	0 (0)	19.6 (11)	0.0026^*^	0 (0)	4.3 (1)	1.0000
Ser83 → Thr	2.6 (1)	0 (0)	0.4043	0 (0)	8.7 (2)	0.4889
Ser83 → Phe + Asp87 → Ala	0 (0)	3.6 (2)	0.5132	0 (0)	0 (0)	—
Ser83 → Leu + Asp87 → Tyr	0 (0)	3.6 (2)	0.5132	0 (0)	0 (0)	—
Ser83 → Tyr	0 (0)	1.8 (1)	1.0000	0 (0)	0 (0)	—
Asp87 → Asn	2.6 (1)	0 (0)	0.4043	0 (0)	0 (0)	—
Ser83 → Leu + Asp87 → His	0 (0)	1.8 (1)	1.0000	0 (0)	0 (0)	—
Ser83 → Phe	0 (0)	0 (0)	—	0 (0)	4.3 (1)	1.0000
No mutation	2.6 (1)	1.8 (1)	1.0000	60.9 (14)	60.9 (14)	1.0000

^*^ *P* < 0.05.
